# Proteomic Analysis of Mesenchymal Stem Cells from Normal and Deep Carious Dental Pulp

**DOI:** 10.1371/journal.pone.0097026

**Published:** 2014-05-08

**Authors:** Dandan Ma, Li Cui, Jie Gao, Wenjuan Yan, Ying Liu, Shuaimei Xu, Buling Wu

**Affiliations:** 1 Department of Stomatology, Nanfang Hospital, Guangzhou, P.R. China; 2 College of Stomatology, Southern Medical University, Guangzhou, P.R. China; Rutgers - New Jersey Medical School, United States of America

## Abstract

Dental pulp stem cells (DPSCs), precursor cells of odontoblasts, are ideal seed cells for tooth tissue engineering and regeneration. Our previous study has demonstrated that stem cells exist in dental pulp with deep caries and are called carious dental pulp stem cells (CDPSCs). The results indicated that CDPSCs had a higher proliferative and stronger osteogenic differentiation potential than DPSCs. However, the molecular mechanisms responsible for the biological differences between DPSCs and CDPSCs are poorly understood. The aim of this study was to define the molecular features of DPSCs and CDPSCs by comparing the proteomic profiles using two-dimensional fluorescence difference gel electrophoresis (2-D DIGE) in combination with matrix-assisted laser desorption ionization time-of-flight mass spectrometry (MALDI-TOF MS). Our results revealed that there were 18 protein spots differentially expressed between DPSCs and CDPSCs in a narrow pH range of 4 to 7. These differently expressed proteins are mostly involved in the regulation of cell proliferation, differentiation, cell cytoskeleton and motility. In addition, our results suggested that CDPSCs had a higher expression of antioxidative proteins that might protect CDPSCs from oxidative stress. This study explores some potential proteins responsible for the biological differences between DPSCs and CDPSCs and expands our understanding on the molecular mechanisms of mineralization of DPSCs in the formation of the dentin-pulp complex.

## Introduction

Human dental stem cells are generally applied in tissue and organ regeneration; however, the regenerative application of these stem cells in dental therapy remains problematic [1]. To date, five types of human dental stem cells have been isolated and characterized: dental pulp stem cells (DPSCs) [2], [3], stem cells from exfoliated deciduous teeth (SHED) [4], stem cells from apical papilla (SCAP) [5], dental follicle stem cells (DFSCs) [6] and periodontal ligament stem cells (PDLSCs) [7], [8].

DPSCs, which are ideal seed cells for tooth tissue regeneration, can differentiate into functional odontoblasts in vivo when the tooth encounters external mild stimuli such as carious lesion, attrition and abrasion. The reactionary and reparative dentin formed by surviving odontoblasts and newly differentiated odontoblast-like cells protect the pulp from further damage. Our previous study has indicated that stem cells exist in carious pulp and are named carious dental pulp stem cells (CDPSCs). CDPSCs displayed an increased proliferative capacity and enhanced alkaline phosphatase (ALP) activity, mineralization ability, and the expression of osteogenesis/dentinogenesis-related genes compared with DPSCs [9]. Though the biological characteristics of these two stem cells have been well analyzed, the molecular mechanisms responsible for the biological differences between CDPSCs and DPSCs are still unclear.

Mass spectroscopy (MS) based proteomics is becoming an efficient method characterized by systematic large-scale qualitative and quantitative mapping of the whole proteome of stem cell phenotypes from different niches, allowing for the rapid understanding the mechanisms that control their self-renewal ability, differentiation potential and regeneration capacity [10], [11].

Previous studies compared the protein expression profiles in mesenchymal stem cells derived from human periodontal ligament, dental pulp, dental follicle, and dental papilla to provide a database for proteins commonly or differentially expressed among various dental stem cell populations [12], [13], [14]. Recently Pivoriuūnas A et al. analyzed the proteomic profiling of SHED to reveal the abundantly expressed proteins [15].

In this work, we performed two-dimensional fluorescence difference gel electrophoresis (2-D DIGE) in combination with matrix-assisted laser desorption ionization time-of-flight mass spectrometry (MALDI-TOF MS) to identify the differentially expressed proteins between DPSCs and CDPSCs and to explore the candidate molecular markers contributing to the regeneration of dental structures in stem cell-based tissue engineering protocols.

## Materials and Methods

### Cell Culture and Identification

All patient-related procedures (patients were 18–20 years of age) used in this study were approved by the Medical Ethics Committee of Nanfang Hospital, and written informed consent was obtained from all subjects. Normal pulp tissues were collected from freshly extracted third molars without caries or pulpal diseases (n = 10). Carious pulp tissues were obtained from wisdom teeth diagnosed with deep caries (n = 10). The diagnosis of deep caries was determined by endodontic specialists according to clinical assessment. Inclusion criteria were the following: carious lesion depth was 80% or more of the dentine thickness assessed radiographically and the presence of a clear radiodense area between the carious lesion and the pulp. The thickness of the remaining dentin was less than 2 mm. Exclusion criteria were: prolonged intense pain, spontaneous pain, and/or pain disturbing a full night’s sleep; apical radiolucency; negative response to thermal and electric pulp testing. All pulp tissues were minced and then digested with 3 mg/mL collagenase type I (Invitrogen Life Technology, Carlsbad, CA, USA) and 4 mg/mL dispase (Sigma, St Louis, MO, USA) for 1 h at 37°C. Single-cell suspensions were obtained by passing the digested tissues through a 70-µm cell strainer (Carrigtwohill Co, Cork, Ireland). The cells were seeded into 6-well plates (Costar, Cambridge, MA, USA) with Dulbecco’s modified Eagle medium (Gibco, Life Technologies, Grand Island, NY, USA) containing 15% fetal bovine serum, 100 units/mL penicillin, 100 mg/mL streptomycin, and 50 mg/mL ascorbic acid and then incubated at 37°C in 5% CO_2_. Stem cells obtained from normal pulp tissues were called DPSCs, and those from carious pulp tissues were called CDPSCs. DPCSs and CDPSCs were enriched by collecting multiple colonies.

### Scanning Electron Microscope Study

DPCSs and CDPSCs at passage 3 were used for the study. The samples were prefixed at 4°C in 2.5% glutaraldehyde overnight and post-fixed in 1% OsO_4_ for 2 h at room temperature (RT). They were dehydrated in gradual increased concentration of ethanol and then critical point dried. The samples were observed under a scanning electron microscope (XL 30 ESEM, Philips Electron Optics, Eindhoven, The Netherlands).

### Cell Counting Assay

Following 24 h serum starvation, DPSCs and CDPSCs were seeded at 2×10^4^ in 1 mL medium per well of a 24-well plate for the cell counting assay. The cell growth medium was replaced every two days. At the indicated time points, cell proliferation was analyzed by counting cells using trypan blue for exclusion of dead cells. The assay was repeated in triplicate.

### In vitro Analysis of Multilineage Differentiation of DPSCs and CDPSCs

For osteogenic differentiation, DPSCs and CDPSCs were seeded in 2 mL complete culture medium at 3×10^4^/35 mm plate and cultured to 70% confluence. Differentiation was induced by culturing cells in complete medium supplemented with 10 mM β-glycerol phosphate, 50 µg/mL ascorbic acid, and 10^−7^ M dexamethasone for 3 weeks. The induced cells were fixed in 70% ice-cold ethanol for 20 min at RT and then stained with 2% alizarin red S. For adipogenic differentiation, DPSCs and CDPSCs were seeded into 24-well plates at a density of 1×10^4^/well and cultured to 70% confluence. Differentiation was induced by culturing cells in complete medium supplemented with 0.5 mM methylisobutylxanthine, 0.5 mM hydrocortisone, and 60 mM indomethacin for 3 weeks. The cells were fixed in 4% paraformaldehyde (PFA) for 20 min at RT and then stained with oil red O. For chondrogenic differentiation, DPSCs and CDPSCs were prepared as described for adipogenic differentiation. The cells were incubated with 50 µg/mL ascorbic acid, 1% insulin-transferrin-selenous acid, 100 mg/mL sodium pyruvate, 40 µg/mL L-proline and 10 µg/L transforming growth factor-3 for 3 weeks. Finally, the cells were then fixed in 4% PFA for 20 min at RT and then stained with alcian blue.

### Protein Determination and 2D-DIGE

Samples for DPSCs and CDPSCs were centrifuged at 1,200 g and washed in ice-cold PBS three times. Total proteins were extracted from cells with 500 µL of lysis buffer (7 M urea, 4% CHAPS, 20 mM Tris, 2 M Thiourea and 2 mM TBP) and a mixture of protease inhibitors. The extraction mixture was sonicated three times for 20 s with 40% amplitude by using U200S sonicator (IKA Labortechnik, Germany) and then centrifuged at 15,000 g for 1 h at 4°C. The Bradford assay was performed to determine the protein content of DPSCs and CDPSCs. All samples were stored at −80°C prior to electrophoresis. 2D-DIGE was performed according to the manufacturer’s protocol (CyDye DIGE Fluor minimal dyes, GE Healthcare) with minor modifications outlined below. For IEF, samples were labeled with three Cy-Dye DIGE fluors (Cy2, Cy3 and Cy5). A total of 50 µg of protein sample was labeled with 400 pmol of Cy3 or Cy5. Cy2 was used to label two mixed samples as an internal reference standard. The samples were vortexed briefly and incubated on ice for 30 min in the dark. Reactions were quenched by the addition of 1 mL of 10 mM lysine and incubated for 10 min on ice under dark conditions. The labeled samples were pooled and mixed with rehydration buffer (7 M urea, 2 M thiourea, 4% CHAPS, 40 mM DTT, 0.00004% bromphenol blue and 0.2% IEF buffer) to a final volume of 450 µL. To ensure an optimal focusing of the proteins, an ampholyte solution for pH 4–7 (Serva, Heidelberg, Germany) was added in a concentration of 1% and the samples were loaded onto Immobiline Dry Strips (IPG, 24 cm, linear pH gradient from pH 4–7, GE Healthcare) by rehydration for 20 h at RT in darkness. The strips were rehydrated and focused with the following parameters: 150 V for 1 h and 250 V for 1 h, respectively; then 1 h at 500 V, 1 h at 1000 V, 5000 V for 3 h by gradient; finally 10000 V for 4 h by step and hold until a total amount of 70000 Vh was obtained with a current limit of 50 µA/gel. Subsequent to IEF, the strips were equilibrated with 10 mg/mL DTT and 40 mg/mL iodoacetamide for 15 min in equilibration buffer containing 6 M urea, 30% glycerol, 2% SDS, and 75 mM Tris-HCl (pH 8.8). Gels were run in Laemmli electrophoresis running buffer (250 mM Tris base, 1.92 M glycine and 1% SDS) and sealed on the borderline of the SDS-PAGE gel by using 0.5% low-melting point agarose gel. Protein spots were separated in 12.5% SDS-polyacrylamide gels at 2 W/gel for 1 h at 16°C and then 17 W/gel at 10°C until the bromophenol blue dye reached the end of the gel. The biological triplicates were run on three gels as analytical gels.

### Image Scanning and Analysis

Three different gel images were performed from one gel at the appropriate wavelengths. They are Cy2 (blue 488 nm laser and 520 nm band pass emission filter), Cy3 (green 532 nm laser and 580 nm band pass emission filter) and Cy5 (red 633 nm laser and 670 nm band pass emission filter) by using a Typhoon 9410 scanner (GE Healthcare) to generate eighteen protein spot maps. DeCyder 5.0 software (GE Healthcare) was used for 2D-DIGE analysis according to the manufacturer’s recommendation. The DeCyder differential in-gel analysis (DIA) module was used for pairwise comparisons of each sample with the internal standard in each gel. The DeCyder biological variation analysis (BVA) module was then used to simultaneously match all nine protein spot maps, using the Cy3/Cy2 and Cy5/Cy2 DIA ratios, to calculate average abundance changes. Student’s t-test was used to calculate significant differences in relative abundances of protein spot features in DPSCs compared with CDPSCs. The differential protein spots (|ratio|>2, p<0.05) were selected for further identification.

### Identification of Protein Spots by MS

Protein spots were cut from gels and washed twice with Milli-Q water, destained with 50% acetonitrile (ACN) and 50 mM ammonium bicarbonate (NH_4_HCO_3_), and dried under vacuum. Each spot was digested overnight in 20 ng/µL trypsin in 40 mM NH_4_HCO3. The peptides were extracted two times with 50% ACN and 5% TFA. The extracts were pooled and dried completely by centrifugal lyophilization. Then, 1 µL of the mixture was loaded onto a target plate with 10 mg/mL CHCA matrix, dried at RT, and analyzed using Voyager DE STR MALDI-TOF (ABI, USA). All analyses were carried out in reflex positive ion mode at an accelerating voltage of 20 kV and a reflex voltage of 23 kV. The instrument was calibrated with external standards including P14R and Insulin Chain B Oxidized. Internal mass calibration was performed using trypsin auto-digestion products. The obtained peptide mass fingerprints (PMF) were submitted for identification using the Mascot search engine (Matrix Science, UK). Search parameters were set as follows: taxonomy human, cysteine acetylation, enzyme trypsin, one missed cleavage site, and a peptide tolerance of 100 ppm.

### Western Blot Analysis

Cells were harvested and homogenized for total protein extraction. Equivalent amounts of protein extracts were separated by SDS-PAGE and then transferred to nitrocellulose membranes (Amersham Biosciences, Buckinghamshire, UK). Rabbit anti-CCT2 (1∶1000; ProteinTech Group Inc, Wuhan, China), anti-stathmin(1∶500; ProteinTech Group Inc), and anti-β-actin antibody (1∶2000; Cell Signaling Technology, Beverly, MA, USA) were used as the primary antibodies and horseradish peroxidase conjugated goat anti-rabbit IgG (Thermo Scientific Pierce, Rockford, IL, USA) was used as the secondary antibody. Immunoreactive bands were detected using SuperSignal (R) West Femto Maximum Sensitivity Substrate (Thermo Scientific Pierce). The analysis was performed three times.

### Statistical Analysis

The data were analyzed and expressed as the mean ±standard deviation. Statistical significance was evaluated by independent samples *t*-test using SPSS Statistics V21.0 software. Statistical significance was set at *P*<0.05.

## Results

### Morphological Analyses

**Figure 1:**
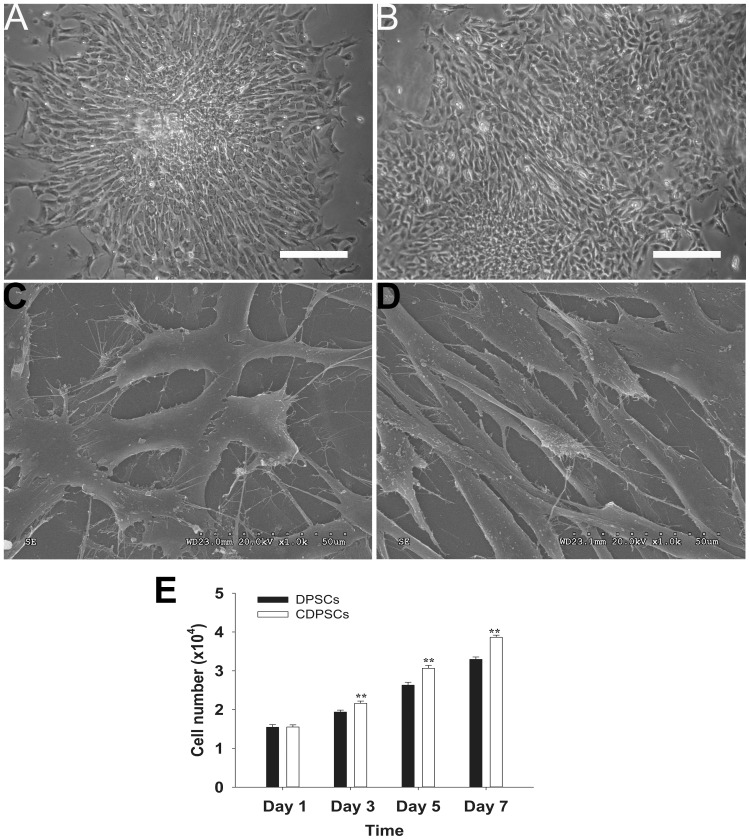
Morphology and proliferative potential of DPSCs and CDPSCs. Both DPSCs (A) and CDPSCs (B) isolated from dental pulp were spindle-shaped and fibroblast-like under the light microscope. Both DPSCs (C) and CDPSCs (D) have a fibroblast-like appearance with long cytoplasmic processes and many filopodia using the scanning electron microscope. (E) CDPSCs have a higher proliferative potential compared with DPSCs (**p<0.01). Scale bar = 100 µm. Each experiment was repeated in triplicate.

We compared DPSCs and CDPSCs at passage 3. Typical colonies of DPSCs and CDPSCs growing in 25 cm^2^ culture flasks for two weeks were identified after seeding as single cell suspensions. Under light microscopy, both stem cell populations formed single colonies in culture. Both DPSCs (Figure 1A) and CDPSCs (Figure 1B) were spindle-shaped and fibroblast-like and the cellular nuclei were round or oval-shaped. The fine structure of both stem cells was studied using scanning electron microscopy. Both DPSCs (Figure 1C) and CDPSCs (Figure 1D) had a morphological homogeneous fibroblast-like appearance with long cytoplasmic processes and many filopodia.

### Cell Growth

The proliferation rates of DPSCs and CDPSCs were studied using the cell counting technique at passage 3. The data showed that the number of DPSCs was significantly lower than that of CDPSCs at days 3, 5, and 7, indicating that CDPSCs had a higher proliferative potential in comparison with DPSCs (**p<0.01) (Figure 1E).

### Multipotent Differentiation

**Figure 2:**
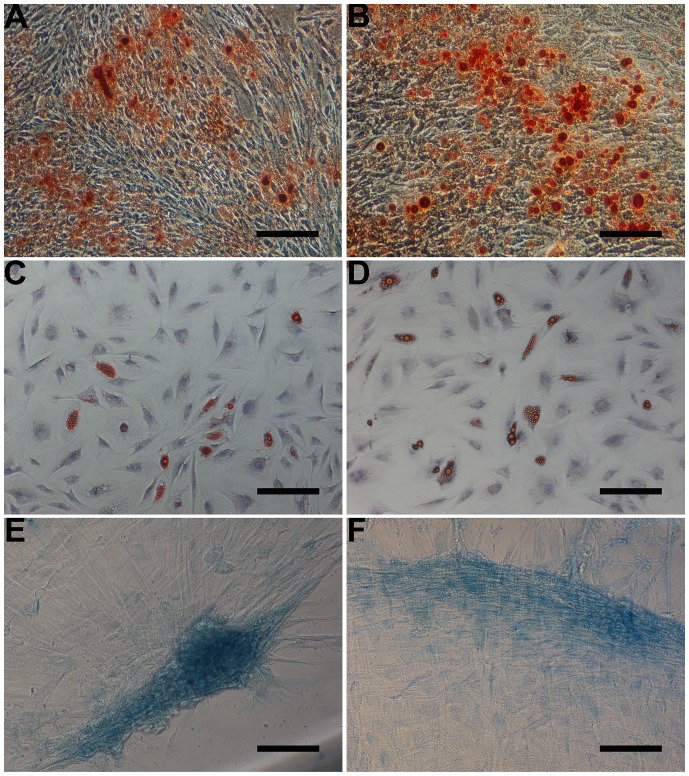
Multilineage differentiation potential of DPSCs and CDPSCs. Mineralization assay in DPSCs and CDPSCs. Mineralized nodules formed by DPSCs (A) and CDPSCs (B) were detected by alizarin red S staining after 3 weeks of culture in mineralized-induced media. Adipogenic differentiation, visualized by oil red O staining, showed lipid vacuoles in DPSCs (C) and CDPSCs (D). Chondrogenic differentiation was visualized by alcian blue staining of DPSCs (E) and CDPSCs (F), demonstrated by the accretion of sulfated matrix. Scale bar = 100 µm. Each experiment was repeated 3 times.

To evaluate the multipotent differentiation capacity, both DPSCs and CDPSCs were treated with various differentiation-inducing media. Osteogenic differentiation was indicated by the detection of mineralized nodules in DPSCs (Figure 2A) and CDPSCs (Figure 2B) after 3 weeks of culture in mineralization medium. Following the induction of adipogenic differentiation, the accumulation of lipid-rich vacuoles in DPSCs (Figure 2C) and CDPSCs (Figure 2D) was visualized within cells by oil red O staining. The induction of chondrogenic differentiation was demonstrated by the accretion of sulfated matrix in DPSCs (Figure 2E) and CDPSCs (Figure 2F) stained with alcian blue.

### DPSCs and CDPSCs Proteome in Narrow pH Range (4–7)

**Figure 3:**
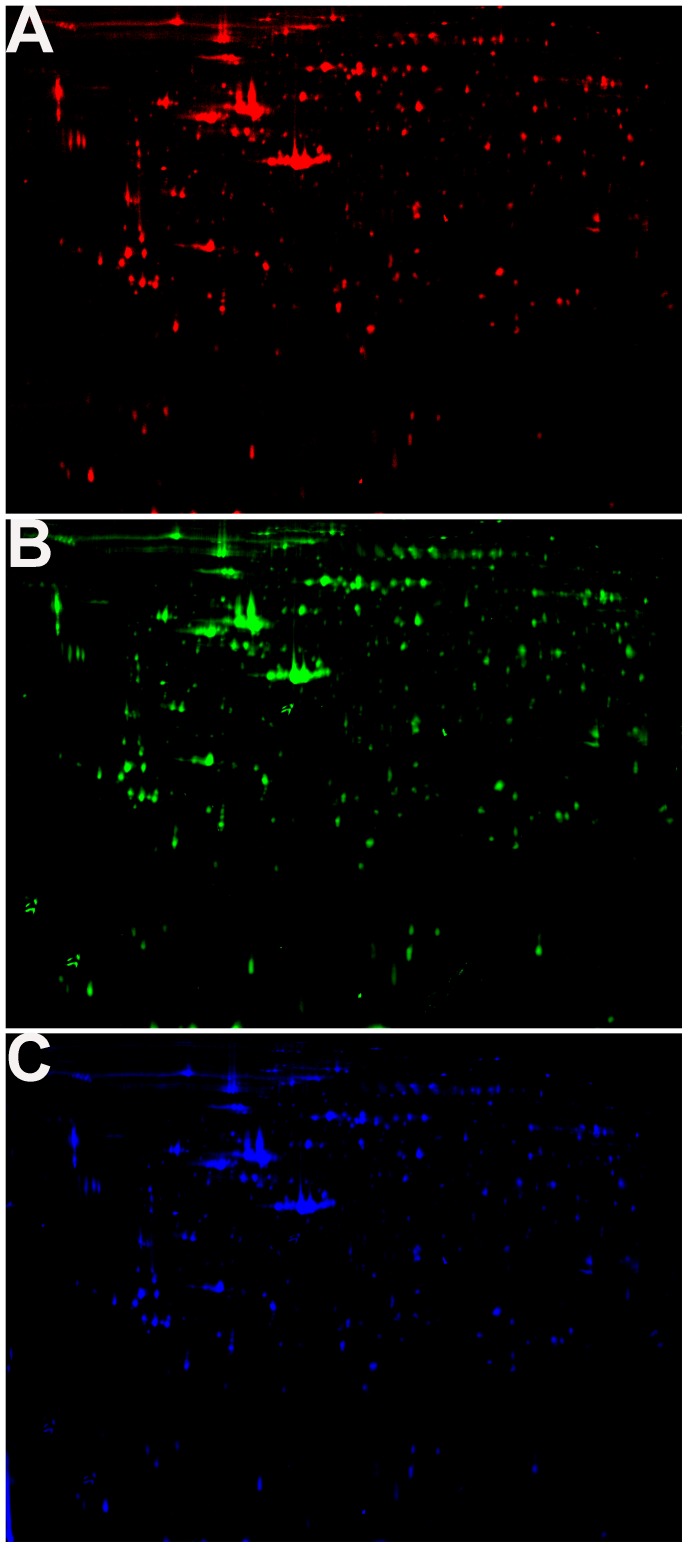
2D-DIGE of DPSCs and CDPSCs. A Cy3 dye staining of CDPSCs B Cy5 dye staining of DPSCs. C Cy2-labeled internal standard proteome map.

**Figure 4:**
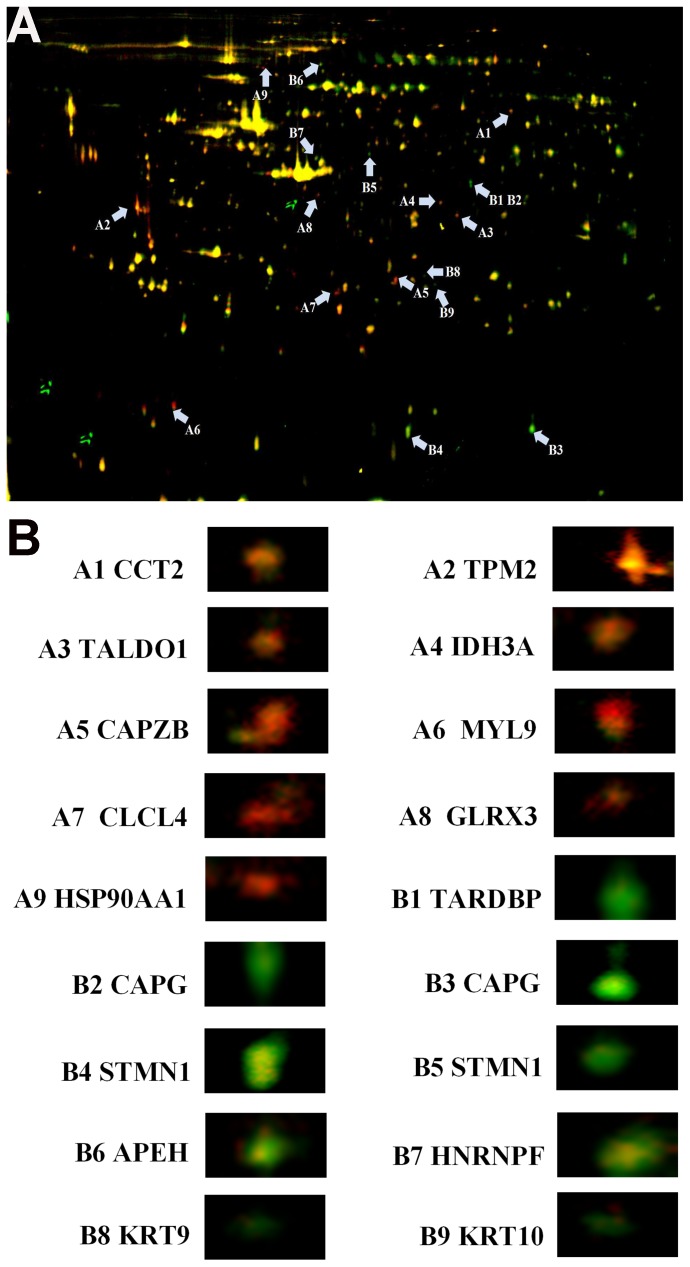
Changes in protein expression between DPSCs and CDPSCs. A The merge of Cy3, Cy5 and Cy2. Distribution of 18 differentially expressed protein spots in fluorescence difference gel electrophoresis gels. Protein spots are indicated (arrows). B Enlarged images of the differentially expressed protein spots in DIGE analysis.

To better understand the differentially expressed proteins of DPSCs and CDPSCs, we analyzed the DPSCs and CDPSCs by 2D-DIGE. Proteins were labeled with Cy3 or Cy5 fluorescent dye, and then the Cy3, Cy5 and Cy2 images were scanned and analyzed using DeCyder 5.0 software (Figure 3). Compared with DPSCs, 18 protein spots were differentially expressed in CDPSCs. Among them, 9 protein spots had increased expression while 9 had reduced expression in CDPSCs (Figure 4). The 18 protein spots were selected for further identification with MALDI-TOF MS. The pH 4–7 range analysis showed that CDPSCs presented a higher expression level of T-complex protein 1 subunit beta (CCT2), tropomyosin beta chain (TPM2), transaldolase (TALDO1), isocitrate dehydrogenase [NAD] subunit alpha, mitochondrial (IDH3A), F-actin-capping protein subunit beta (CAPZB), myosin regulatory light polypeptide 9 (MYL9), chloride intracellular channel protein 4 (CLIC4), glutaredoxin-3 (GLRX3), heat shock protein HSP 90-alpha (HSP90AA1) and DPSCs presented a higher expression level of TAR DNA-binding protein 43 (TARDBP), macrophage-capping protein (CAPG), stathmin (STMN1), acylamino-acid-releasing enzyme (APEH), heterogeneous nuclear ribonucleoprotein F (HNRNPF), keratin, type I cytoskeletal 9 (KRT9), keratin, type I cytoskeletal 10 (KRT10) (Table 1). All identified proteins were analyzed using the online GOfact interface (http://61.50.138.118/gofact). These proteins were potentially related to cell proliferation, differentiation, cell cytoskeleton and motility, and antioxidant function. They are widely distributed in the cytoplasm, nucleus, membrane and mitochondria (see support information, Table S1).

**Table 1:** Differentiated expressed proteins between DPSCs and CDPSCs.

Spot	Entrynames	Protein names	AccessionNo.(Swiss-Prot)	TheoreticalMW/pI	Summaryscore
A1	CCT2	T-complex protein 1 subunit beta	P78371	57794/6.02	68
A2	TPM2	Tropomyosin beta chain	P07951	32945/4.66	91
A3	TALDO1	Transaldolase	P37837	37688/6.36	55
A4	IDH3A	Isocitrate dehydrogenase [NAD] subunit alpha,mitochondrial	P50213	40022/5.71	111
A5	CAPZB	F-actin-capping protein subunit beta	P47756	31616/5.36	148
A6	MYL9	Myosin regulatory light polypeptide 9	P24844	19871/4.78	108
A7	CLIC4	Chloride intracellular channel protein 4	Q9Y696	28982/5.45	128
A8	GLRX3	Glutaredoxin-3	O76003	37693/5.31	87
A9	HSP90AA1	Heat shock protein HSP 90-alpha	P07900	85006/4.94	64
B1	TARDBP	TAR DNA-binding protein 43	Q13148	44711/5.85	36
B2	CAPG	Macrophage-capping protein	P40121	38494/5.82	29
B3	CAPG	Macrophage-capping protein	P40121	38494/5.82	29
B4	STMN1	Stathmin	P16949	17292/5.76	83
B5	STMN1	Stathmin	P16949	17292/5.76	83
B6	APEH	Acylamino-acid-releasing enzyme	P13798	82142/5.29	85
B7	HNRNPF	Heterogeneous nuclear ribonucleoprotein F	P52597	45985/5.37	105
B8	KRT9	Keratin, type I cytoskeletal 9	P35527	62255/5.14	80
B9	KRT10	Keratin, type I cytoskeletal 10	P13645	59046/5.13	75

### Western Blot

**Figure 5:**
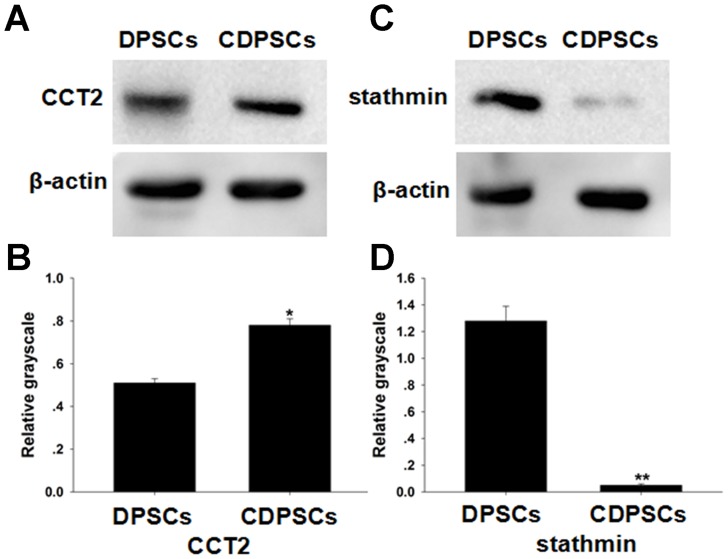
Altered expressions of two identified proteins. The expression level of the CCT2 protein was lower in DPSCs than CDPSCs (A, B). DPSCs have a higher expression level of stathmin protein compared with CDPSCs (C, D). The change in protein expression was consistent with that of proteomic analysis (*p<0.05, **p<0.01). Each experiment was repeated 3 times.

We chose one upregulated protein and one downregulated protein in CDPSCs to compare with DPSCs. The interestingly selected identified proteins CCT2 and stathmin, which are closely correlated with cell proliferation and differentiation, were confirmed by western blot, the results demonstrated that CDPSCs had a higher expression of CCT2 and a lower expression of stathmin compared with DPSCs, which suggested that the proteomic analyses based on 2-D DIGE were convincing (Figure 5).

## Discussion

Recent studies have reported that DPSCs are able to differentiate into various cell types or tissues including osteoblasts [3], odontoblasts [16], chondroblasts [3], [17], adipocytes [17], neuronal cells [18], endothelial cells [19], melanocytes [20] and cornea [21]. Among these, the most important function of DPSCs is forming odontoblasts, however; the mechanisms that are responsible for DPSCs migration, proliferation, and differentiation when the tooth encounters deep caries are poorly known.

Our study revealed that both DPSCs and CDPSCs had fibroblast-like morphology and were shown to be capable of differentiating into various cell types including osteoblasts, adipocytes and chondrocytes. Moreover, CDPSCs had a higher proliferative potential than DPSCs, which was consistent with the previous study [9].

To better understand the molecular mechanisms underlying the changes in DPSCs encountering deep caries, we used 2D-DIGE to identify the proteins differentially expressed between DPSCs and CDPSCs. The comparative narrow range PH analysis showed that most differentially expressed proteins between the two stem cell populations are potentially related to cell proliferation, differentiation, cell cytoskeleton and motility, and antioxidative function. These differentially expressed proteins may contribute to the biological differences between CDPSCs and DPSCs.

A group of differentially expressed proteins are closely related to cell proliferation and differentiation including CCT2, stathmin and CLIC4. Chaperonin containing t-complex polypeptide 1 (CCT) is essential for maintaining cellular homoeostasis by assisting the folding of many proteins such as cytoskeleton proteins, actin and tubulin. CCT is composed of eight different subunits (1, 2, 3, 4, 5, 6, 7, and 8) and their functions are poorly understood [22]. Previous studies showed that CCT2 expression was important for normal cell proliferation [23], [24]. Moreover, CCT2 is overexpressed in certain malignant tumors and its overexpression is closely correlated with poor prognosis [25]. CCT2 also involves in the regulation of the processes of cellular motion and neuronal differentiation [26], [27]. As CCT2 is a potential positive regulator of cell growth, the up-regulation of CCT2 in CDPSCs may be partly responsible for the observation that CDPSCs have a higher proliferation capacity compared with DPSCs.

CLIC4 is ubiquitously expressed in almost every cell type studied and is found in transmembranes and intramembranes. It is implicated in diverse cellular processes including membrane trafficking, cell proliferation, cell-cycle control, cell differentiation and morphogenesis [28], [29]. CLIC4 is essential for keratinocyte survival and is involved in the regulation of endothelial proliferation [29], [30]. It is closely related to adipocyte and keratinocyte differentiation [29], [31]. In addition, recent studies also provide evidence that the down-regulation of CLIC4 impairs angiogenesis and tubular morphogenesis [32]. CLIC4 is also shown to play a role in immune response of macrophages to LPS and in the host defense against bacterial infection [33]. As CLIC4 is a positive regulator of cell proliferation, differentiation and host defense, microbial products produced by bacteria might enhance the expression of CLIC4 in CDPSCs in the case of deep caries. The up-regulation of CLIC4 might be accountable for the increased proliferation and osteogenic differentiation capacity of CDPSCs compared with DPSCs.

Stathmin is a ubiquitous cytosolic regulatory phosphorprotein and is involved in diverse intracellular signaling pathways including cell proliferation, cell-cycle regulation, differentiation, microtubule dynamics and activities [34], [35]. Up- and down regulation of stathmin has similar inhibitory effect on cell proliferation by interfering with the formation and dynamics of mitotic spindles responsible for cell mitosis and the normal cell cycle [36]. Recent studies showed that stathmin promoted osteoblast differentiation and bone mass formation by interfering with microtubule assembly [35]. The expression level of stathmin diminishes in all cells detected so far as they become more terminally differentiated in culture [37]. CDPSCs under deep caries stimulation have greater osteogenic capacity compared with DPSCs, suggesting that CDPSCs might be more differentiated than DPSCs; thus, stathmin is expressed higher in DPSCs than in CDPSCs. The essential role of stathmin in regulating the cytoskeleton microtubules indicated that it may be required for the biological functions of DPSCs.

Another group of differentially expressed proteins is correlated with cell cytoskeleton and motility and includes TPM2, MYL9, CAPZB, CAPG, KRT9 and KRT10. Tropomyosins (TPM) are a family of actin-filament binding proteins expressed in most eukaryotic cells. In human, there are at least four TPM genes (TPM1, TPM2, TPM3 and TPM4). TPM2 is found primarily in skeletal muscles and this protein helps regulate muscle contraction by interacting with other muscle proteins, particularly myosin and actin [38]. In addition, TPM2 is essential for cytoskeleton establishment and the regulation of TGF-β induced stress fiber formation [39].

MYL9 can be phosphorylated by myosin light chain kinase in the presence of calcium and calmodulin. This phosphorylation contributes to the increase in the actin-activated ATPase activities of myosins. MYL9 is involved in the regulation of both smooth muscle and nonmuscle cell contractile activity via its phosphorylation [40]. Moreover, phosphorylation of MYL9 alters myocardium contraction by increasing the force and rate of force development [41].

CAPZB is a heterodimer with an α subunit of 32–36 kDa and a β subunit of 28–32 kDa. The actin barbed end capping protein is highly conserved and found in nearly all eukaryotic cells [42]. CAPZB regulates the assembly of actin filament structures which are required for numerous biological processes and precise coordination.

CAPG is a member of the gelsolin-villin family, and it binds to actin in a calcium-dependent manner. CAPG is wildly distributed in tissues and cells. Previous studies have shown that its function is closely correlated with the motility and ruffling of various cell types including macrophages, neutrophils, fibroblasts, and endothelial cells [43], [44], [45]. Recently, CAPG was reported to be an essential protein for the embedding and dendrite elongation processes in osteocytes [46].

KRT9, a type I intermediate filament protein, is abundant in human foot soles and palms. In mice, KRT9 is a major component of the perinuclear ring of manchette in spermatids and is required for normal sperm development [47], [48]. Mutation of KRT9 is responsible for human epidermolytic palmoplantar keratoderma and degenerative changes of keratin’s intermediate filament structure [49].

KRT10, a type I keratin protein, is normally expressed in the suprabasal epidermal compartment. Deletion of KRT10 impairs permeability barrier function and stratum corneum hydration [50]. KRT10 is required for epidermal integrity, as KRT10 mutation leads to epidermolytic hyperkeratosis [51].

In this study, 3 identified proteins were mainly related to antioxidative function including TALDO1, GLRX3, and APEH. TALDO1 is an enzyme of the pentose phosphate pathway. TALDO1 deficiency has been implicated in a widening spectrum of diseases including male infertility, acetaminophen-induced acute liver failure, cirrhosis, hepatocellular carcinoma and autoimmunity diseases [52]. NADPH and GSH protect cells against oxidative stress. The effect of TALDO1 depletion on NADPH and GSH is discordant among various cells. Suppression of TALDO1 increases NADPH and GSH production and enhances oxidative stress in human Jurkat and H9 T cells, however; TALDO1 deficiency diminishes NADPH and GSH production in human lymphoblasts [53], [54], [55]. The up-regulation of TALDO1 in CDPSCs may increase NADPH and GSH expression, which may contribute to protecting CDPSCs from oxidative damage.

GLRX3, an essential [2Fe–2S]-binding protein, has been reported to play important roles in various signaling pathways including embryogenesis, immune cell response, the regulation of cardiac hypertrophy, cancer cell functions and iron homeostasis [56], [57], [58]. GLRX3 is necessary to protect cells against oxidative stress and GLRX3 deletion leads to embryonic lethality in mice [59], [60]. The pulp with deep caries is more likely to suffer from oxidative stress. The up-regulation of GLRX3 in CDPSCs may help protect them from being damaged by reactive oxygen species.

APEH belongs to the prolyl oligopeptidase family of serine proteases. Previous studies have showed that APEH contributes to the elimination of the oxidized proteins in mammalian cells and in plants [61], [62]. APEH may be a potential regulator in sustaining the homeostasis of the cytoplasmic antioxidative system. The expression of APEH should be enhanced in CDPSCs, as dental pulp with deep caries is more susceptible to oxidized stress; however, our proteomic analysis showed opposite results. The antioxidative role of APEH in CDPSCs requires further investigation.

HSP90AA1 is a highly conserved and abundant protein. It has been implicated in the activation of various proteins such as important mediators of signal transduction, cell cycle regulation, differentiation and pathogenic factors involved in tumor progression [63]. Previous studies showed that HSP90AA1 played an important role in infectious disease by interacting with various bacterial and viral proteins [64], [65]. Moreover, HSP90AA1 is unregulated under elevated temperature [66]. Two reasons may be accountable for the up-regulation of HSP90AA1 in CDPSCs. First, the by-products secreted by bacteria and viruses might enhance the expression level of HSP90AA1 in stem cells isolated from dental pulp undergoing stimulation from a deep carious lesion. Moreover, the fluctuating oral temperature may increase HSP90AA1 expression in CDPSCs, as dental pulp with deep caries is more vulnerable to temperature stimuli without sufficent hard dental tissue protection.

Isocitrate dehydrogenase catalyzes the oxidative decarboxylation of isocitrate to form a-ketoglutarate. This process is considered to be one of the key enzymes in the tricarboxylic acid (TCA) cycle. In mammals, there are three types of isoenzymes represented by cytosolic NADP-specific IDH (IDH1), mitochondrial NADP-specific IDH (IDH2), and mitochondrial NAD-specific IDH (IDH3) [67]. IDH3 is composed of three distinct types of subunits in the ratio 2alpha:1beta:1gamma [68]. The function of the alpha subunit sequence is highly conserved among the mammalian species. Moreover, it contains the isocitrate binding site and is indispensable for catalytic activity [69], [70].

TARDBP and HNRNPF are both important for gene regulation. TARDBP is normally concentrated in the nucleus but also shuttles between the nucleus and cytoplasm. TARDBP plays an important role in the regulation of splicing, microRNA processing, mRNA transport, stability, and translation. Recent studies showed that TARDBP knockdown inhibited neurite outgrowth and causes cell death [71]. TARDBP dysfunction has been linked to neurological disorders, such as amyotrophic lateral sclerosis (ALS), frontotemporal lobar dementia (FTLD) and Alzheimer’s disease (AD) [72].

Heterogeneous nuclear ribonucleoprotein F (HNRNPF) is a member of the HNRNP family that is essential in splicing events. It plays a vital role in modulating gene expression at the transcriptional and posttranscriptional levels. Previous studies have showed that HNRNPF participates at various steps in processing cellular mRNA [73], [74], [75].

In conclusion, we revealed some candidate proteins that might be responsible for the biological differences between CDPSCs and DPSCs. The differently expressed proteins between DPSCs and CDPSCs are mostly involved in the regulation of cell proliferation, differentiation, cell cytoskeleton and motility. In addition, our results suggested that CDPSCs in dental pulp with deep caries have a higher level of expression of antioxidative proteins that may protect CDPSCs from oxidative stress. Further studies are warranted to elucidate the role of potential candidate proteins that may favor dental tissue regeneration.

## Supporting Information

Table S1 **The cellular distribution, molecular function and biological process of the differentiated expressed proteins.**
(DOC)
