# Supplementary material for: Proteomic Analysis of Mesenchymal Stem Cells from Normal and Deep Carious Dental Pulp
Source: PLoS One. 2014 May 8;9(5):e97026. doi: 10.1371/journal.pone.0097026 (PMC4014579; doi:10.1371/journal.pone.0097026)
Supplement: Table S1 — The cellular distribution, molecular function and biological process of the differentiated expressed proteins. (DOC) [file pone.0097026.s001.doc]

**Table S1 The cellular distribution, molecular function and biological process of the differentiated expressed proteins.**

| **Protein names** | **Cellular distribution** | **Molecular function** | **Biological process** |
| --- | --- | --- | --- |
| T-complex protein 1 subunit beta | cytoplasm | [ATP binding](http://www.ebi.ac.uk/QuickGO/GTerm?id=GO:0005524); nucleotide binding; unfolded protein binding | [protein folding](http://www.ebi.ac.uk/QuickGO/GTerm?id=GO:0006457); cellular protein metabolic process |
| Tropomyosin beta chain | [cytosol](http://www.ebi.ac.uk/QuickGO/GTerm?id=GO:0005829); cytoskeleton; cytoplasm;  [muscle thin filament tropomyosin](http://www.ebi.ac.uk/QuickGO/GTerm?id=GO:0005862) | [actin binding](http://www.ebi.ac.uk/QuickGO/GTerm?id=GO:0003779); structural constituent of muscle | muscle contraction; [muscle filament sliding](http://www.ebi.ac.uk/QuickGO/GTerm?id=GO:0030049); regulation of ATPase activity |
| Transaldolase | cytoplasm | [catalytic activity](http://www.ebi.ac.uk/QuickGO/GTerm?id=GO:0003824) | [carbohydrate metabolic process](http://www.ebi.ac.uk/QuickGO/GTerm?id=GO:0005975) |
| Isocitrate dehydrogenase [NAD]  subunit alpha, mitochondrial | [mitochondrion](http://www.ebi.ac.uk/QuickGO/GTerm?id=GO:0005739); mitochondrial matrix | [NAD binding](http://www.ebi.ac.uk/QuickGO/GTerm?id=GO:0051287); oxidoreductase activity; isocitrate dehydrogenase (NAD+) activity; metal ion binding | carbohydrate metabolic process; tricarboxylic acid cycle; oxidation-reduction process; cellular metabolic process; small molecule metabolic process |
| F-actin-capping protein subunit beta | cytoplasm; intercalated disc; cytosol; WASH complex; membrane; F-actin capping protein complex; Z disc; sarcomere; acrosomal vesicle; actin cytoskeleton; lamellipodium; cortical cytoskeleton | actin binding; actin filament binding; beta-tubulin binding | microtubule polymerization regulation; cellular component movement; cytoskeleton organization; cell projection organization; lamellipodium assembly; blood coagulation; regulation of cell morphogenesis; muscle fiber development; barbed-end actin filament  capping; actin filament capping; regulation of protein kinase C signaling; actin cytoskeleton organization |
| Myosin regulatory light polypeptide 9 | cytosol; stress fiber; myosin complex; muscle myosin complex; Z disc; myosin II complex | structural constituent of muscle; calcium ion binding; metal ion binding | muscle contraction; axon guidance; regulation of muscle contraction |
| Chloride intracellular channel protein 4 | cytoplasm; cytosol; [apical part of cell](http://www.ebi.ac.uk/QuickGO/GTerm?id=GO:0045177); mitochondrion; plasma membrane; centrosome; microvillus; microtubule organizing center; microtubule cytoskeleton; midbody; intracellular; nucleus; [cell surface](http://www.ebi.ac.uk/QuickGO/GTerm?id=GO:0009986); [nuclear matrix](http://www.ebi.ac.uk/QuickGO/GTerm?id=GO:0016363); chloride channel; cell-cell junction; membrane; cytoplasm; [Z disc](http://www.ebi.ac.uk/QuickGO/GTerm?id=GO:0030018); cell cortex cell-cell junction; membrane; complex; integral component of membrane; [cytoplasmic vesicle membrane](http://www.ebi.ac.uk/QuickGO/GTerm?id=GO:0030659); cytoplasmic actin cytoskeleton | voltage-gated ion channel activity; [chloride channel activity](http://www.ebi.ac.uk/QuickGO/GTerm?id=GO:0005254); voltage-gated chloride channel activity; protein binding | [angiogenesis](http://www.ebi.ac.uk/QuickGO/GTerm?id=GO:0001525); [fertilization](http://www.ebi.ac.uk/QuickGO/GTerm?id=GO:0009566); ion transport; [keratinocyte differentiation](http://www.ebi.ac.uk/QuickGO/GTerm?id=GO:0030216); [vacuolar acidification](http://www.ebi.ac.uk/QuickGO/GTerm?id=GO:0007035); regulation of anion transport; [cellular response to calcium ion](http://www.ebi.ac.uk/QuickGO/GTerm?id=GO:0071277); endothelial cell morphogenesis; [apical/basal cell polarity](http://www.ebi.ac.uk/QuickGO/GTerm?id=GO:0035088) regulation; cell differentiation; negative regulation of cell migration; ion transmembrane transport; multicellular organism growth; branching morphogenesis of epithelial tube; [multicellular organism growth](http://www.ebi.ac.uk/QuickGO/GTerm?id=GO:0035264) |
| Glutaredoxin-3 | cytoplasm; [Z disc](http://www.ebi.ac.uk/QuickGO/GTerm?id=GO:0030018); cell cortex | [electron carrier activity](http://www.ebi.ac.uk/QuickGO/GTerm?id=GO:0009055); [metal ion binding](http://www.ebi.ac.uk/QuickGO/GTerm?id=GO:0046872); [iron-sulfur cluster binding](http://www.ebi.ac.uk/QuickGO/GTerm?id=GO:0051536); protein binding; protein disulfide oxidoreductase activity; protein kinase C binding | regulation of the force of heart contraction; regulation of cardiac muscle hypertrophy; metabolic process; cell redox homeostasis; oxidation-reduction process |
| Heat shock protein HSP 90-alpha | extracellular region; apical plasma membrane; intracellular; cytoplasm; cytosol; plasma membrane; cell surface; basolateral plasma membrane; brush border membrane; melanosome; neuron projection; neuronal cell body; perinuclear region of cytoplasm; endocytic vesicle lumen protein complex; mitochondrion | nucleotide binding; ATPase activity; UTP binding; CTP binding; ATP binding; GTP binding; mRNA binding; protein binding; sulfonylurea receptor binding; protein kinase binding; dATP binding protein phosphatase binding; nitric-oxide synthase regulator activity; TPR domain binding; identical protein binding; protein homodimerization activity; ion channel binding; unfolded protein binding | mitotic cell cycle; regulation of cardiac muscle contraction; regulation of cell size; neuron migration; skeletal muscle contraction; ATP catabolic process; protein folding; mitochondrial transport; response to stress; response to unfolded protein; axon guidance;  signal transduction; response to salt stress; response to heat; chaperone-mediated protein complex assembly;  nitric oxide metabolic process; regulation of nitric-oxide synthase activity; innate immune response; protein import; small molecule metabolic process; response to estrogen; regulation of lamellipodium assembly; cardiac muscle cell apoptotic; Fc-gamma receptor signaling |
| TAR DNA-binding protein 43 | [nucleus](http://www.uniprot.org/locations/SL-0191) | protein binding; sequence-specific DNA binding transcription factor activity; nucleotide binding | DNA-templated transcription; RNA splicing; transcription from RNAP II promoter; mRNA processing; cell death; regulation by host of viral transcription; mRNA stabilization |
| Macrophage-capping protein | nucleus | actin binding | cell projection assembly |
| Stathmin | intracellular; cytoskeleton; cytoplasm; cytosol; membrane; microtubule | signal transducer activity; tubulin binding | microtubule polymerization and depolymerization axonogenesis; nervous system development; cell differentiation; multicellular organismal development;  mitotic spindle organization; response to virus signal transduction; cellular component movement regulation |
| Acylamino-acid-releasing enzyme | cytoplasm; nuclear membrane | serine-type peptidase activity | [proteolysis](http://www.ebi.ac.uk/QuickGO/GTerm?id=GO:0006508) |
| Heterogeneous nuclear  ribonucleoprotein F | nucleoplasm; spliceosomal complex; cytoplasm; nucleus; ribonucleoprotein complex | nucleotide binding; protein binding | RNA processing; RNA splicing; gene expression; mRNA splicing, via spliceosome; regulation of RNA splicing |
| Keratin, type I cytoskeletal 9 | [intermediate filament](http://www.ebi.ac.uk/QuickGO/GTerm?id=GO:0005882) | [structural molecule activity](http://www.ebi.ac.uk/QuickGO/GTerm?id=GO:0005198) | [spermatogenesis](http://www.ebi.ac.uk/QuickGO/GTerm?id=GO:0007283) |
| Keratin, type I cytoskeletal 10 | [cytoplasm](http://www.ebi.ac.uk/QuickGO/GTerm?id=GO:0005737); [intermediate filament](http://www.ebi.ac.uk/QuickGO/GTerm?id=GO:0005882); [keratin filament](http://www.ebi.ac.uk/QuickGO/GTerm?id=GO:0045095) | structural molecule activity; structural constituent of epidermis | [cellular response to calcium ion](http://www.ebi.ac.uk/QuickGO/GTerm?id=GO:0071277); epithelial cell differentiation; cellular response to calcium ion |
